# Spatial Characteristics of the Diffusion of Residential Solar Photovoltaics in Urban Areas: A Case of Seoul, South Korea

**DOI:** 10.3390/ijerph18020644

**Published:** 2021-01-13

**Authors:** Moon-Hyun Kim, Tae-Hyoung Tommy Gim

**Affiliations:** 1 Graduate School of Environmental Studies, Seoul National University, Seoul 08826, Korea; moonhyun@snu.ac.kr; 2 Graduate School of Environmental Studies, Interdisciplinary Program in Landscape Architecture, and Environmental Planning Institute, Seoul National University, Seoul 08826, Korea

**Keywords:** peer effects, diffusion of innovation, residential solar photovoltaic, renewable energy, urban area

## Abstract

Mini-solar photovoltaics, which are installed on apartment balconies, are rapidly spreading in Seoul, South Korea. Seoul has implemented a policy to diffuse mini-solar photovoltaics in apartments for energy transition since 2012. The policy considers compact land use and a large population of the city. This study examines a variety of variables in relation to the adoption of mini-solar photovoltaics. In particular, we focus on peer effects, namely, those of spatially adjacent, previously installed mini-solar photovoltaics. As apartment characteristics, four variables are selected to assess both within and between apartment complexes: one for the density of adopters as a within-complex variable and three for the number of adopters in the 500 m, 1 km, and 1.5 km radius of apartment complexes as between-complex variables. A major finding is that peer effects significantly contribute to the new adoption of mini-solar photovoltaics. Implications of this finding are discussed in an urban context.

## 1. Introduction

Reducing the contribution to atmospheric carbon dioxide is a challenging task and renewable energy is a major determinant to deal with it. The atmospheric concentration of carbon dioxide (CO_2_), which is one of nine planetary boundaries (PBs), is considered an indicator to identify the risk of climate change [1]. Steffen et al. [2] warned that more than 450 ppm is beyond the high-risk area, which could be potentially devastating to the resilience system of Earth. Thus, increasing attention has been focused on renewables, which do not emit CO_2_ in generating electricity, to reduce CO_2_ emission in an effort to mitigate climate change. Among various renewable energy sources, concerns with household-scale electricity generation are centered on solar photovoltaics (PVs).

Numerous studies were conducted to find what drives social diffusion in the residential adoption of universal rooftop PVs [3,4,5]. Most studies investigated smaller town residents who usually live in single-family houses, and others were based on multi-family buildings of a larger city focused on rooftop PVs [6]. Only a few studies exist on residential PVs in an urban area. Thus, this study differs in that it examines household solar PVs installed on apartment balconies in a megacity region. It aims to obtain an expanded understanding of the spatial characteristics of residential solar PVs in an urban area with a multitude of apartment complexes, such as Seoul, South Korea.

As the national capital, Seoul leads an effort to expand the generation of renewable energy in South Korea. Since 2012, “One Less Nuclear Power Plant” has become a representative energy policy of Seoul [7]. According to deeper citizen participation, the policy attempts to reduce energy consumption and increase electricity self-sufficiency by using renewable energy. In particular, Seoul declared the policy of “Solar City Seoul, 2022” in 2017, which intends to distribute solar panels to one million households (65% for mini-solar PVs installed on apartment balconies and 35% for rooftop PVs) by 2022.

At this juncture, with the case of Seoul, this study aims to analyze the adoption and diffusion of mini-solar PVs using annual panel data of 2014–2017. This empirical analysis focuses on peer effects while controlling for demographic, economic, built environment, and socio-political variables. It specifically has three objectives: (i) to typify the distribution pattern of the mini-solar PVs, which tends to be dispersed from neighboring previous adoptions; (ii) to explain the characteristics of apartment complexes with mini-solar PVs; and (iii) to investigate the proximity between new and previous adoptions in relation with peer effects “within” and “between” apartment complexes. This study will eventually provide implications for explaining the applicability of the renewables and spatial characteristics to diffuse them in an urban context.

## 2. Literature Review

Rogers [8] argues that common rules are found among those who accept innovations, that is, diffusion of innovation occurs through the communication channel for the generation and sharing of information among the members of a similar social system. Rai and Henry [9] highlight the importance of communication strategies with the rate and scale of adoption. In this respect, the individual’s behavior was found to be under the control of the social norm, which has been called social interaction, peer effect, imitation behavior, and herd behavior in the literature [5,10].

Regarding the diffusion of residential solar PVs (RSPVs), previous studies focused on the identification of the characteristics of the space in which peer effects are present and on the discovery of which attributes affect residential adoption. Bollinger and Gillingham [3] were the first to confirm the peer effect of adoption of RSPVs. They found that in California, 0.78% of the possibility of a new adoption in a ZIP code is raised by one additional installation in that area. They further argued that the addition has a greater effect on larger households and commuters of more than a 30-minute distance. Regarding the peer effect, household size and commuting distance both increase the chance to see solar PV installations on the street.

Several UK studies analyzed the peer effect using data on solar PV adoption on the postal code scale. Richter [11] reported that the social interaction effect is significant, albeit modest, and Snape [12] presented that areas with similar solar PV installation capacity are clustered in space and the adoption rate increases over time. Likewise, in Germany, Dharshing [5] conducted spatial regression and confirmed clustering at the county level.

From a different perspective, some studies analyzed how the distance between neighborhoods influence the adoption of solar PVs. Graziano and Gillingham [13] conducted a panel analysis of a time series dataset of solar PV adoptions in Connecticut to evaluate how the spatial distribution for RSPVs is affected according to the buffer distances of 0.5 miles or less, 0.5–1 mile, and 1–4 miles at the census block group level. They found that about 0.44 PV systems on average are added by a new installation within 0.5 miles, suggesting the peer effect. Müller and Rode [14] analyzed the physical distance between the locations of solar PVs using a binary panel logit model in Wiesbaden, Germany. The distance exerted a strong negative effect on the social interaction. Rode and Weber [15] examined the peer effect through an epidemic diffusion model based on a dataset of RSPVs for 576,000 households in Germany. They selected raster widths of 0.5 km, 1 km, 4 km, and 10 km to explore the spatial effect and subsequently found that the effect is significant in the distance of 1 km and a larger variation is explained when the model considers spatial homogeneity.

While the adoption of solar PVs has been studied, focusing on spatial distribution, some of the studies may further expand our understanding at the individual/household level. Rai and Robinson [16] specified multiple regression models based on survey data of RSPV adopters to analyze perception, informational gaps, and technological uncertainties. They tested various hypotheses regarding PV adopters’ decision-making and found the significant effects of neighboring PV installations, experiences with leased products, and contacts with previous adopters of RSPVs. A later study by Rai and Robinson [4] developed an agent-based model of RSPV adoption and analyzed a household-level dataset of PV adopters in Texas. As a result, social networks between homogeneous individuals or groups were found to strongly affect each other. Recently, Rai and Henry [9] recommended an agent-based model as a tool for analysis of the complex energy demand in relation to social influences and spatial constraints.

## 3. Methodology

### 3.1. Study Area: Mini-Solar Photovoltaics in Seoul

**Figure 1:**
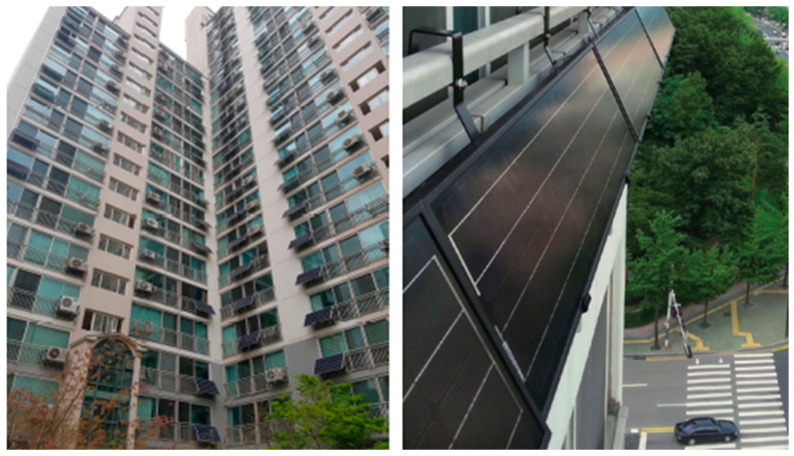
Mini-solar photovoltaics (PVs). Source: Seoul Metropolitan Government, Seoul Sustainable Energy Action Plan [17].

**Figure 2:**
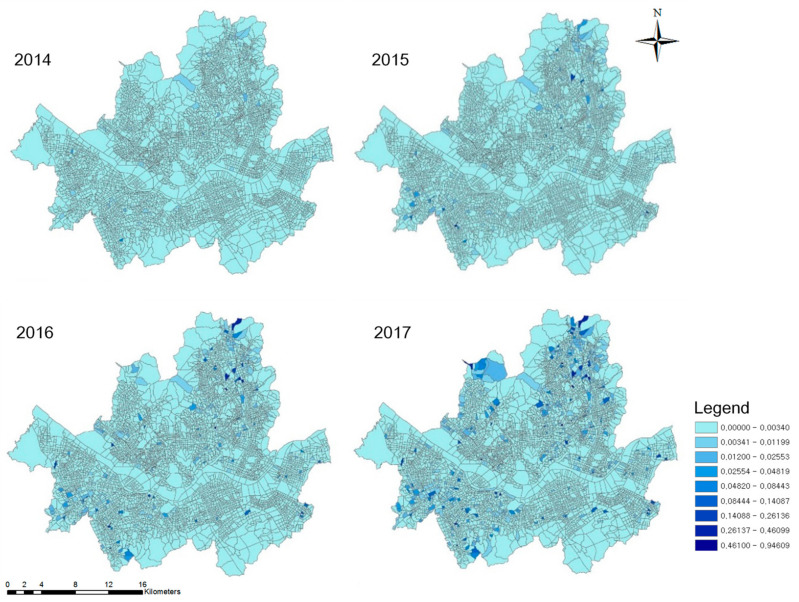
Density of mini-solar PVs at the block group level.

**Figure 3:**
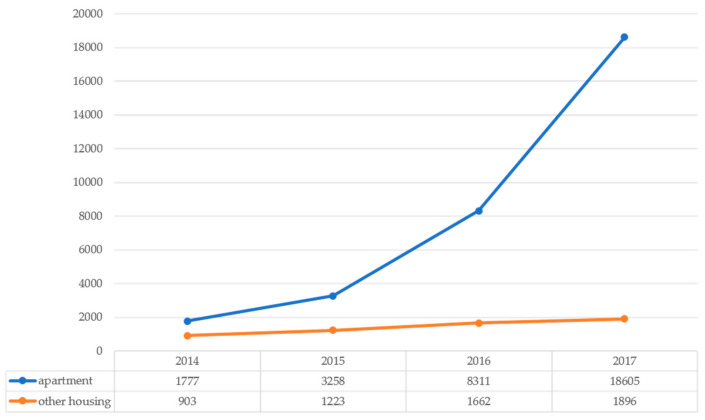
Number of households with solar PVs by housing type in Seoul.

Since 2012, Seoul has implemented the policy of “One Less Nuclear Power Plant” to proactively approach the energy crisis and climate change. A notable goal of the first phase of this policy was to expand the solar energy [17]. Solar panels were installed not only in public institutions and schools, but also in private buildings and houses. Introduced to Seoul in 2013, mini-solar PVs (the power range from 50 W to 1 kW) for apartments are widely used because they have a compact module and inverter, in comparison to the existing house rooftop solar panels, and they can be easily installed in a small space in apartment balconies (Figure 1). Electricity can be generated and consumed for the daily needs of the household. As shown in Figure 2 and Figure 3, apartment mini-solar PVs are still in their infancy but are continuously distributed.

Seoul is developing efforts to expand mini solar PVs as a way of achieving 20% electricity self-sufficiency in 2020 in comparison to 5% in 2014 [17]. By 2022, mini-solar PVs will be installed in 605,185 apartment balconies. Anyone who intends to install mini-solar PVs receives an average of 364,000 KRW (about 330 USD) and 420,000 KRW (about 380 USD) of financial support (more than 60% of the total cost) for 260 and 300 watts of installed capacity, respectively, from the Seoul Metropolitan Government, along with the district governments [18]. Furthermore, the invested capital is likely to be paid back at 260 W in 2.3 years and 300 W in 2.9 years, which has been assessed to be economically feasible [19].

The unit of analysis of this study is apartment complexes built before 2014 in Seoul. The data were obtained from the website of the Korean Management System for Multi-family Housing, called K-apt. This study analyzed 795 apartment complexes (706,836 households) across Seoul by excluding those cases with missing values. They represent continuous panel data between 2014 and 2017.

### 3.2. Dependent Variable

The dependent variable was the number of new adopters of RSPVs in the sample of 795 apartment complexes each year. We geocoded RSPVs that were installed in Seoul from 2014 to 2017 at the apartment complex level. The count dependent was characterized by discrete, not normal, distribution, only with positive integers. Thus, if one employs ordinary least squares (OLS) regression to analyze these discrete count data, the OLS estimate will be biased [20]. This justified the specification of a Poisson-family regression model. Specifically, data with a mean of 3.92 and a variance of 288.3 denoted over-dispersion. As such, instead of Poisson regression, negative binomial regression was preferred [21].

**Figure 4:**
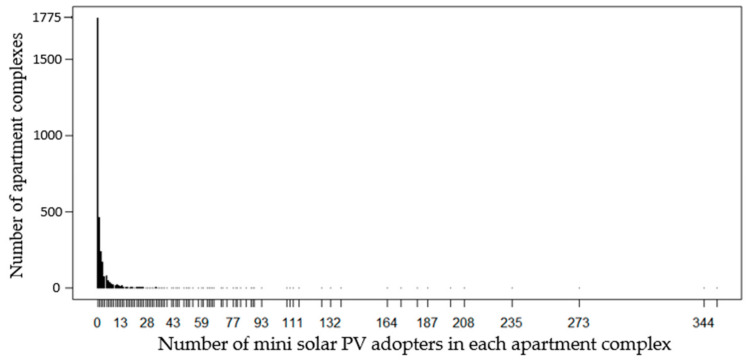
Distribution of mini-solar PVs in Seoul.

Figure 4 shows the number of apartment complexes by that of mini solar PV adopters in each apartment complex. The total apartment complexes were 3180 (= 795 complexes × 4 years). The total number of apartment complexes without mini-solar PVs were 1775, accounting for about 55.82% of the total. In this case of excessive zero values, a zero-inflated model (i.e., zero-inflated negative binomial regression) appeared to be appropriate.

### 3.3. Independent Variables

#### 3.3.1. Demographic Variables

Studies reported differing effects of age on the adoption of RSPVs. Parkins et al. [22] argued that age has a negative effect on adoption intention, which is related to environmental value and political participation. Meanwhile, a marketing study [3] presented a lower adoption rate among those aged 20–45 and over 65. Similarly, others concluded that, among those in their 40s, the adoption rate is higher [23,24] or more insignificant [13]. A Sri Lankan study [25] presented a positive relationship between middle age (40–49) and the adoption of RSPVs and argued that, in their 30s or younger, people cannot afford the initial cost of the installation. While no particular studies are found based on the case of Seoul, this study categorized ages into groups according to population ratios through a dasymetric mapping method [26,27] (population ratio by age group of apartment complexes). In particular, ages were classified into 20–39, 40–69, and equal to or more than 70 years.

#### 3.3.2. Socio-Political Variables

As socio-political variables, we chose the political affiliation of the elected head of the district government (Seoul consists of a total of 25 district governments), the membership of the district government in the ICLEI (Local Governments for Sustainability organization), and its participation in the energy community program (run by the Seoul Metropolitan Government). Determined by the political party of the government head, the political affiliation is believed to measure the willingness to supply RSPVs and reflect citizens’ beliefs and values. Studies [28,29] presented the tendency for democrats to be equipped with higher environmental values and for areas, whose leaders are from the democratic party, to have more installations of RSPVs [22]. Additionally, the pro-government sentiment affects the adoption of residential solar PVs in Malta at the initial stages of the plan [30].

In fact, a regression study [23] suggested that the ICLEI membership may not be significant in the delivery/installation of RSPVs, mainly because the membership does not strongly require actions regarding climate change and sustainable development. In a South Korean (hereafter, Korean) study [31], however, the membership of the district government was found to significantly reduce energy consumption, suggesting practical actions for the climate change mitigation. As such, we employed the ICLEI membership variable. The member list was obtained from the ICLEI Korea website. An apartment complex had a value of 1 if it was a part of the ICLEI member and 0 otherwise.

In Seoul, the participation of the energy community program is considered among the critical determinants of the installation of RSPVs [32]. Once enrolled in this program, households receive financial incentives as a return for proven information on energy reduction activities. The program accordingly encourages households to install RSPVs. Energy communities steadily increased to 58 communities in 2017 (15 in 2014, 29 in 2015, and 45 in 2016).

#### 3.3.3. Economic Variables

This study examined how subsidization encourages the installation of RSPVs. Provided by Seoul and its 25 district governments, financial incentives are known as a major determinant of the adoption of RSPVs [32,33]. As of 2017, all of the district governments offer this subsidy (only 1 in 2014, 5 in 2015, and 14 in 2016). Similarly, the revenues and costs mainly affect the decision of installation for residential solar PVs [34]. Meanwhile, a recent study [35] suggested that the decision factors to uptake RSPVs differ between earlier and later adopters. Earlier adopters tend to be motivated by non-financial factors, such as environmental concern, whereas later adopters are driven by economic gains.

Previous studies suspected that the adoption of RSPVs is associated with income: the higher the income, the more likely the household is to install solar PVs [36]. However, recent studies [3,14] found that low income households have a stronger tendency to install solar PVs. This study subsequently investigated how household income affects the adoption of RSPVs using the variables of electricity consumption and the unit price of the property. As for these particular variables, Rodriguez-Oreggia and Yepez-Garcia [37] found that household income has a positive effect on energy consumption, whereas Soytas et al. [38] argued that the effect is insignificant. This study measured the mean electricity consumption by year per apartment complex.

#### 3.3.4. Built Environment Variables

Snape [12] suspected that the low capacity of PV installations is associated with the residential density. That is, densely populated urban areas with high rise buildings including apartments have a limited capacity relative to suburban and rural areas, which is possibly because a major city with a large population density has limited space to install solar PVs. To test this, this study chose the following built environment variables (per apartment complex): number of houses, mean living area, and building age.

Notably, public rental apartments (in the form of affordable housing) owned by the government were not considered. This was because, since 2015, several district governments mandatorily installed mini-solar PVs in rental apartments in accordance with an order of their heads. Since this study focuses on the social interaction among the residents of the apartment complexes, the mandatory installation may have misestimated the peer effect. In addition, due to data limitations, this study did not include built environment-related technical variables that may affect the adoption of mini solar PVs; however, the orientation and potential capacity of mini solar PV differentiate the installation conditions.

#### 3.3.5. Peer Effect Variables

This study hypothesized the social interaction to be influenced by neighboring RSPVs in the apartment complex. According to social normative theory, people tend to follow social norms since they are influenced by the behavior or attitudes of others [39]. According to this theory, we considered two types of effects, word-of-mouth and visibility, to evaluate the social interaction. Word-of-mouth is caused by neighbors who have had a good impression of RSPVs and recommend them to others. Friendship, advice, and support within social networks facilitate the innovation of adoption and diffusion [40]. Thus, RSPVs are suspected to diffuse within the apartment complex where people actively interact through various community activities. Second, Arkesteijn and Oerlemans [41] developed the idea that the adoption of innovative technology is associated with visual and social effects. Indeed, the visual exposure to solar technology has been found to have a positive effect on the spreading of RSPVs [3,22,42].

Notably, we considered the possible distance in which the social interaction occurs. Regarding the distance, previous studies tested multiple buffers. Graziano and Gillingham [13] examined how many households adopted solar systems within the 0.5-mile, 1-mile, and 4-mile radii of each installation and found the 0.5-mile buffer to be significant. Rode and Weber [15] set the distance ranges of 0.5 km, 1 km, 4 km, and 10 km and argued that the variable of the 1 km range has a positive effect on the diffusion of the solar system. Rai and Robinson [4] chose the intervals of 30.5 m (100 ft) up to 3050 m (10,000 ft) and, based on a geographical network model, found that the distance of 610 m (2000 ft) has a significant impact.

**Figure 5:**
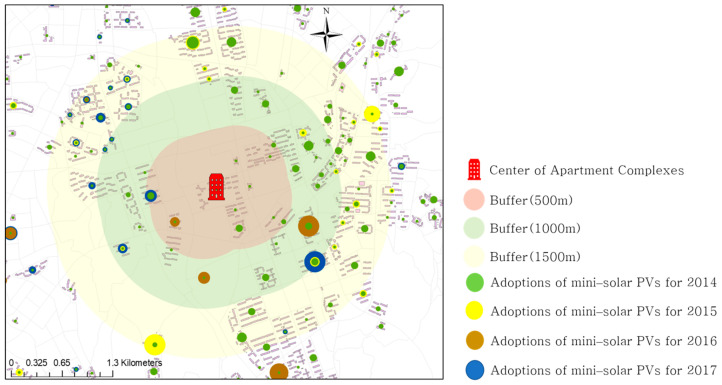
Extraction of the number of households within multi-buffers.

This study set four variables to consider the peer effect “within” and “between” apartment complexes. First, the installation density of RSPVs was calculated to reflect the within-complex peer effect. The value of the variable for 2014 was zero because the business of mini-solar PVs started in 2014. The value for 2015 was the density of mini-solar PVs adopted in 2014 (and those for 2016 and 2017 referred to cumulative densities). In order to capture between-complex peer effects, we designed multi-buffers of the 500 m, 1000 m, and 1500 m radius bands. The number of RSPV installations was extracted within each buffer using ArcGIS, as shown in Figure 5.

### 3.4. Statistical Analysis

The dependent variable had discrete over-dispersed values with excess zeros. Thus, zero-inflated negative binomial (ZINB) regression models were run using R statistical analysis software (R Foundation for Statistical Computing, Vienna, Austria). Our dataset was a strongly balanced panel data from 2014 to 2017, and one can control correlated unobservable errors by using fixed or random effects at the individual level and time level. Apartment complex fixed effects can control endogenous group formation, leading to the self-selection of peers (homophily), and time dummies were employed to reduce unobserved heterogeneity over time [3,13]. This can overcome homophily, correlated unobservables, and simultaneity, which should be considered to identify spatial peer effects [13]. All analyses were performed in R using the open access glmmTMB package [43].

Empirical analyses proceeded in three steps. The initial model analyzed the total sample. We then compared two groups of ten district governments: The first group was based on apartment complexes participating in the energy community program and the second was not. Finally, we examined the effect of clustering (hot/cold spots) based on the density of mini-solar PV installations in Seoul and, accordingly, we compared hot and cold spots, each of which consisted of five district governments.

## 4. Results

### 4.1. Descriptive Statistics

Table 1 presents the descriptive statistics of all research variables. They appear to have enough variations for statistical inference. Table 2 shows the annually new and accumulated adopters of mini-solar PVs. The mean of the new adopters increased to 5.79 in 2017 from 0.74 in 2014, denoting about an eight-time increase in 3 years. The accumulated number of households also increased by 12.8 times. The mean electricity consumption of each apartment complex qA log 2.572 (converted to 373.25 kWh) and was higher than the mean monthly household electricity consumption: 305 kWh in 2014, 304 kWh in 2015, and 309 kWh in 2016 [44]. As for peer effects, the numbers of adopters within multi-buffers were as follows: 20.86 adopters in the 500 m band, 49.55 in the 1000 m band, and 85.62 in the 1500 m band.

### 4.2. Results Based on the Entire Sample

**Figure 6:**
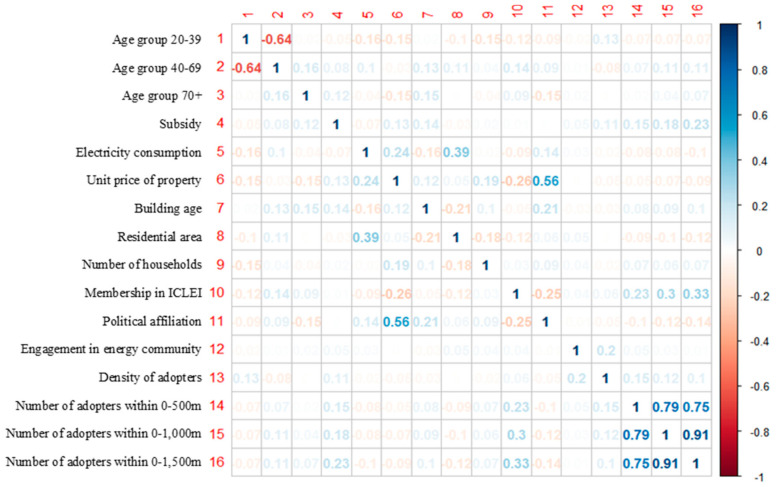
Correlation matrix for independent variables.

Table 3 presents the overall results of Model 1, which was based on the entire sample. Due to multicollinearity, the model excluded the variable of the number of adopters in the 0–1500 m radius (Figure 6).

As a demographic characteristic, age was classified into the groups of 20–39, 40–69, and equal to or more than 70 years. The last 70+ age group was significant. Among the economic variables, subsidy from district governments, mean electricity consumption in the apartment complex, and the unit price of the property turned out to be significant. The subsidy by district governments also positively affected PV adoption. Lastly, the lower the mean electricity consumption and the unit price of the property, the more mini-solar PVs tended to be installed.

Regarding socio-political variables, for apartment complexes participating in the energy community program, the rate of mini-solar PVs installations increased by 12.5 times (≈ e^2.526). Additionally, district governments whose leaders were affiliated with the democratic party, entitled the “Democratic Party of Korea”, tended to achieve more mini-solar PVs in their apartment complexes.

Among the built environment variables, younger apartment complexes were equipped with more mini-solar PVs. Additionally, the greater the number of houses in the complex, the more mini-solar PVs it was likely to be equipped with.

As for peer effects, the density of households with mini-solar PVs had a positive impact on the number of households with new installations. The model also showed a significantly positive coefficient for the 500 m radius band. Meanwhile, for a step width of 0–1000 m the coefficient turned out to be negative, suggesting that the number of adopters sharply reduced for a distance between 500 and 1000 m. That is, in Seoul, mini-solar PVs installed in apartment complexes were clustered at a radius of about 500 m.

### 4.3. Energy Community

The Seoul energy community program comprises various activities for increasing energy citizenship of its residents, which was hypothesized to encourage them to adopt mini-solar PVs. In order to test this hypothesis, this study compared differences in analytical results between a group of 10 district governments with apartment complexes participating in the program and the other 10 not participating. Models 2 and 3 are based on the first and second groups, respectively.

As such, this study found that peer effects were quite different between the two groups. Model 2 presented similar results with Model 1. The density of households with mini-solar PVs and the number of adopters within the 500 m radius band had a positive effect on the new adoption. A 1% increase of the density of adopters increased the possibility of the adoption by 22%, and one adopter increase within the band led to the adoption by 0.16%. In contrast, based on Model 3, while the density of adopters had a positive effect on the new adoption, the number of adopters within the 1000 m radius band had a negative effect. This implies that districts without apartment complexes participating in the energy community program may be capable of delivering mini-solar PVs within each of the apartment complexes but the between-complex effect within the 500 m radius is not significant, while, if the radius exceeds 500 m, the adopters may rather decrease in number.

### 4.4. Hot/Cold Spots

In Table 3, Models 4 and 5 show analytical results based on Local Moran’s *I* to identify a spatial clustering of mini-solar PVs based on the density of adopters by block group in 2017. For local indicators of a spatial associated (LISA) relationship, we employed a spatial weight matrix according to the inverse distance spatial relationship, which was statistically significant, since the value of Local Moran’s *I* was 0.0186 (*p* < 0.001). A positive value denotes that clustering exists.

**Figure 7:**
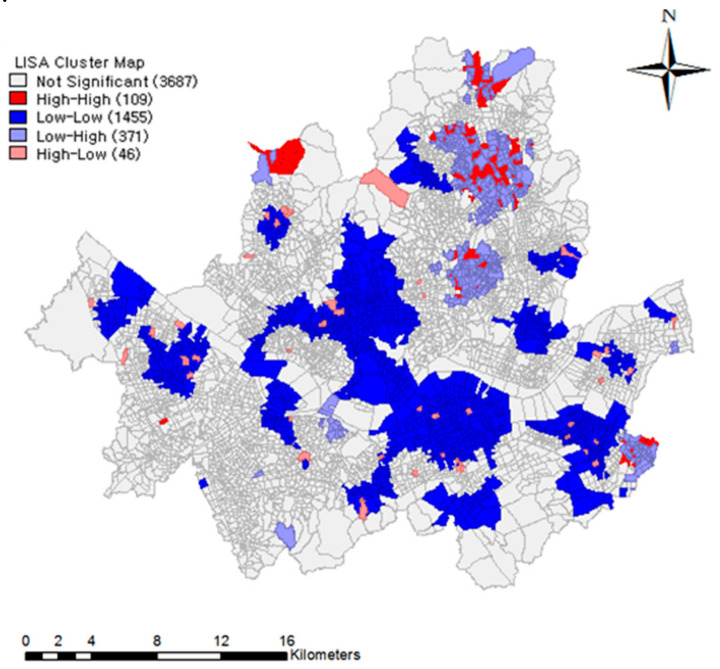
Local Moran’s I: Hot and cold spots of mini-solar PVs.

A clustering of hot spots was present in the northern (Dobong and Nowon Districts), northwestern (Eunpyeong District), and northcentral (Dongdaemun and Seongbuk Districts) parts of Seoul. In comparison, cold spots were largely distributed in the middle section of the city, particularly in the south side (Gangnam and Seocho Districts) and north side (Jongro, Jung, and Yongsan Districts) of the Han River. Figure 7 shows the clustering of hot and cold spots in terms of the density of mini-solar PVs in Seoul. In this regard, we specified two models, each of which consisted of apartment complexes in the hot and cold spots.

Based on the hot spot subsample, Model 4 delivered quite different results from the other models. The variables of the 40–69 age group, the residential area, and the ICLEI membership were newly found to increase the adoption of mini-solar PVs. Additionally, apartment complexes that engaged in the energy community program had an 11.76-times higher demand for mini-solar PVs than those that did not engage. Regarding peer effects, a 1% increase in the density of adopters raised the new adoption rate by 20.66% and one adopter increase of the number of adopters increased by 0.13%. Notably, the peer effect was not significant in Model 5, that is, on apartment complexes in cold spots. The density of adopters also turned out to be insignificant.

In both models, the incentive, unit price of the property, and number of households were found to be significant. A 10,000 Korean won (approximately 10 USD) increase in incentives resulted in a higher possibility of PV adoption by 23.4% and 17.93% for Models 4 and 5, respectively. On the other hand, the unit price of the property was negatively associated with mini-solar PVs: Every 1,000,000 Korean won reduction in the apartment complex land price led to a 26.21% and 0.054% increase in mini-solar PVs for Models 4 and 5, respectively.

## 5. Discussion

Seoul declared the policy of “Solar City Seoul, 2022”, which aims at achieving “solar power capacity of 1 GW” and “solar powered houses of 1 million” by 2022. As part of this policy, Seoul plans to supply mini-solar PVs to apartments, specifically to 605,185 households by 2022 (an apartment is a dominant type of housing in this hyper-dense city). Currently, incentives for the mini-solar PV installation cover over 60% of the total cost, which will eventually impose a considerable burden on the local and city governments. For this reason, alternative policies have been suggested and studied to reduce the subsidy, but at the same time, concerns were raised, since the reduction may hamper the spread of mini-solar PVs [18,19]. At this juncture, focusing on peer effects, this study investigated different types of variables that possibly affect the delivery of mini-solar PVs.

Regarding age, a low proportion of the 70+ age group caused a higher adoption rate of mini-solar PVs, echoing the findings of Kellstedt et al. [45] and Mills and Schleich [46]. Additionally, the 40–69 age group was affected by the adoption of mini-solar PVs in hot spot areas. Likewise, in the previous studies [23,24,25], residents in their 40s were found to have a higher tendency to install PVs. While the potential for the electricity generation of rooftop solar PVs is the highest, from 12 to 2 pm [47], and the electricity cannot be saved and should be consumed immediately, elderly people and middle-aged full-time homemakers tend to stay longer during the daytime. Thus, this age-related result may suggest a better way of yielding more household cost savings through customized PV policies.

The mean electricity consumption and the unit price of the property (a proxy for the household’s income) had negative effects on mini-solar PV installations. Indeed, recent studies [3,14] argued that low-income households are more likely to install solar systems. Possibly, affluent households have a lower interest in mini-solar PVs because they can afford energy costs.

Peer effects turned out to be meaningful in distributing mini-solar PVs, which extends the findings of several previous studies [3,13,14,15,48]. Janssen and Jager [49], which found that an observability of innovation has a positive impact on the adoption of green products and those who have already accepted a technology have a higher tendency to accept new technologies. In relation to social desires, consumers feel satisfied by consuming the same product as their neighbors and occasionally participating in social comparison and imitation. If the density of mini-solar PVs is high in their neighborhood, people may regard mini-solar PVs as a socially acceptable or commonly applied norm and subsequently decide to install the PVs. Frederiks et al. [39] called this a normative social influence.

In terms of the mini-solar PV distribution, peer effects have been identified “within” and “between” apartment complexes. The “within” effect was stronger. This may be because interactions between neighbors usually occur in the unit of the apartment complex, including energy community, as suggested in this study. These interactions would encourage non-adopters to install mini-solar PVs through word-of-mouth. With regard to the participation in the energy community, in Models 2 and 3, each of which consisted of 10 districts with and without apartment complexes in the energy community program, mini-solar PV adopters were clustered within a 500 m radius in areas in which most apartment complexes engage in the program, while a cluster was not formed in areas without such a complex. This implies that the participation in the energy community program assumes a more important role in spreading mini-solar PVs, which is in line with a finding on the peer effect “within” apartment complexes. That is, the word-of-mouth effect is likely to be stronger than the visual effect, supporting a mixed methods study conducted in Sweden [50].

Regarding the visual effect, peer effects on the installation of mini-solar PVs were significant within a radius of 500 m between apartment complexes. The distance of peer effects appears to differ by region: 804 m (0.5 mile) in Connecticut [13], 1 km in Germany [15], and 610 m in Texas [4]. That is, the distance was found to be shorter with the case of Seoul, partially because, in this hyper-compact city, a neighborhood with a certain number of households is usually defined as a shorter/smaller distance/area.

Meanwhile, membership in ICLEI was significant only in Model 4, which used a hot spot subsample. Notably, as shown in Table 3, the model did not control the effect of the political affiliation. Thus, this suggests that the ICLEI membership has an effect, albeit modest, on mini-solar PV adoption in those areas.

Younger apartments were more likely to have mini-solar PVs. While the average life span of mini-solar PVs is currently 15–20 years, the longer they are used, the higher their economic benefits. Thus, those living in older apartments may be less interested in installing mini-solar PVs, since the apartments could be shortly reconstructed and about 100 dollars are required to relocate mini-solar PVs. In Seoul, if the age of an apartment complex reaches 30 years, it can be officially reconstructed, which is arguably among the most profitable financial investments. In this sense, it may be desirable to make it financially and/or technologically easier to transfer mini-solar PVs.

## 6. Conclusions

This study aimed at analyzing different factors on the adoption of mini-solar PVs in apartment complexes in Seoul. Particularly, we hypothesized that peer effects occur around apartment complexes in which mini-solar PVs are actively installed. Peer effects are exerted in the form of word-of-mouth by innovative early adopters and the visual effects of mini-solar PVs that are installed nearby. In particular, a social desire to gain satisfaction by following the innovative behavior of neighbors is considered the main driver for installing the PVs when their PV adoption rate is high and the PVs are installed in adjacent complexes. In order to evaluate these peer effects, this study employed four variables: the density of households with mini-solar PVs up to the preceding year in each apartment complex and the number of adopters within the 500 m, 1000 m, and 1 500m radius bands.

The major findings are that more mini-solar PVs could be installed in an apartment complex if the proportion of aged residents (age 70+) is low and the mean property value is low and when the district head is politically affiliated with the Democratic Party of Korea. Additionally, peer effects turned out to be significant: Specifically, the within-complex effect (word-of-mouth effect) is likely to be stronger than the between-complex effect (visual effect) in an urban context. Regarding the visual effect, new installations were positively affected by the density of previous mini-solar PVs and within a 500 m radius of those complexes in which mini-solar PVs are installed.

Participation in the energy community program was another factor on the mini-solar PV installation. The program consists of practical activities for energy education and conservation. Thus, it possibly increased the awareness of energy citizenship and positively affected the adoption of mini-solar PVs.

The Korean government helps to improve the regional electricity self-sufficiency by expanding the distributed generation, such as small-scale solar PVs. In 2017, the proportion of the distributed generation was only 12% but is planned to be expanded to 30% by 2040 [51]. Mini-solar PV installation in apartment complexes can be used as a means for electricity self-sufficiency in urban areas. It allows for the production of electricity in residential areas and raises the resident’s energy consciousness. While public subsidies for the apartment PV installation are soon to be exhausted, local governments are recommended to facilitate social interaction/peer effects for the purpose of introducing new adopters of mini-solar PVs in their jurisdictions.

## Figures and Tables

**Table 1:** Descriptive statistics.

Variables		Unit	N	Mean	S.D.	Min	Max	Source
**Dependent variable**								
Number of yearly new adopters		Households	3180	2.374	12.641	0	348	Ministry of the Interior and Safety (MOIS)
**Independent variables**								
Demographic variables	Age group 20–39	%	3180	28.45	4.553	10.377	69.301	National Geographic Information Institute, administrated by the Ministry of Land, Infrastructure and Transport (MOLIT)
	Age group 40–69	%	3180	44.382	2.755	22.641	62.585	
	Age group 70+	%	3180	8.118	2.752	0	26.128	
Economic variables	Subsidy	10,000 won	3180	4.23	4.48	0	10	Lee [18]
	Electricity consumption	kWh (log)	3180	2.572	0.128	2.095	3.373	Electronic Architectural Administration Information System (E-AIS), administered by MOLIT
	Unit price of property	Million won (log)	3180	1.292	0.159	0.971	1.938	Seoul Real Estate Information Plaza, administered by the Seoul Metropolitan Government
Built environment variables	Building age	Year	3180	19.564	7.725	2	46	Korean Management System for Multi-family Housing (K-apt), administered by MOLIT
	Residential area	m^2^ (log)	3180	2.029	0.197	0.413	4.435	
	Number of households	100 households	3180	8.8258	7.502	1.56	56.78	
Socio-political variables	Membership in ICLEI	0 or 1	3180	0.295	0.456	0	1	ICLEI Korea
	Political affiliation	0 or 1	3180	0.209	0.407	0	1	Each of the 25 district governments of Seoul
	Engagement in energy community	0 or 1	3180	0.01	0.1	0	1	Seoul Information Communication Plaza, administered by the Seoul Metropolitan Government
Peer effects	Density of adopters	Adopters/ 100 households	3180	0.196	1.225	0	32.08	MOIS
	Number of adopters within 0–500 m	Adopters	3180	20.862	72.866	0	975	
	Number of adopters within 0–1000 m	Adopters	3180	49.549	143.02	0	2005	
	Number of adopters within 0–1500 m	Adopters	3180	85.615	207.96	0	2158	

**Table 2:** Adopters of mini-solar PVs engaging in the energy community program.

Descriptions	Type	Frequency (Average Value) for All Apartment Complexes in Seoul			
		2014	2015	2016	2017
Adoption of mini-solar PVs	New	591 (0.74)	790 (0.99)	1565 (1.97)	4603 (5.79)
	Accumulated	591 (0.74)	1381 (1.74)	2946 (3.71)	7549 (9.50)
Engaging in energy community		4	6	8	18

**Table 3:** Zero-inflated negative binomial regression on the adoption of mini-solar PVs.

	Variables	Model 1	Model 2	Model 3	Model 4	Model 5
Demographic variables	Age group of 20–39	−0.00958	−0.0246	0.02264	0.01169	−0.07498 *
		(0.01282)	(0.01726)	(0.02785)	(0.02291)	(0.04422)
	Age group of 40–69	0.00559	−0.02244	0.05165	0.0715 **	−0.09577
		(0.02265)	(0.03111)	(0.05062)	(0.03518)	(0.09395)
	Age group of 70+	−0.03566 **	−0.0795 ***	−0.0736 *	0.02897	−0.0799
		(0.0173)	(0.02427)	(0.03773)	(0.02771)	(0.06408)
Economic variables	Incentive	0.1832 ***	0.1869 ***	0.1764 ***	0.2103 ***	0.1649 ***
		(0.01338)	(0.02227)	(0.03729)	(0.02203)	(0.05891)
	Electricity consumption	−1.521 ***	−1.573 **	−1.7858 *	−0.5624	−1.419
		(0.4817)	(0.7212)	(0.93297)	(0.7108)	(1.738)
	Unit price of the property	−3.188 ***	−2.625 ***	−3.203 ***	−2.291 **	−8.367 ***
		(0.4351)	(0.7)	(1.08599)	(1.12)	(1.808)
Build environment variables	Year	−0.0526***	−0.0397***	−0.0527***	−0.0397***	−0.0167
		(0.00698)	(0.01034)	(0.01529)	(0.01256)	(0.02142)
	Residential area	0.5014	0.7869	−0.3311	0.9506**	0.02804
		(0.3172)	(0.5798)	(0.68414)	(0.4063)	(1.075)
	Number of households	0.0823 ***	0.05918 ***	0.119 ***	0.0865 ***	0.0634 **
		(0.00517)	(0.0081)	(0.01082)	(0.00769)	(0.02756)
Socio-political variables	Membership in ICLEI	0.08625	0.03974	–0.12208	0.3608 **	0.5036
		(0.1019)	(0.1533)	(0.22449)	(0.1497)	(0.8353)
	Political affiliation	−0.6378 ***	−0.8554 ***	−1.0574 ***	―	−0.01315
		(0.1407)	(0.2117)	(0.31912)	―	(0.6386)
	Engagement in energy community	2.526 ***	2.811 ***	―	2.465 ***	2.303 *
		(0.3797)	(0.4423)	―	(0.4976)	(1.289)
Peer effect variables	Density of adopters	0.231 ***	0.1991 **	0.49 **	0.1878 ***	−0.1352
		(0.07478)	(0.08974)	(0.1964)	(0.06882)	(0.413)
	Number of adopters within 0–500 m	0.001694 *	0.00159 *	0.00413	0.00131 *	−0.00562
		(0.00088)	(0.00088)	(0.00673)	(0.0007)	(0.00809)
	Number of adopters within 0–1000 m	−0.0007 *	−0.0006	−0.00747**	−0.000136	0.003303
		(0.00038)	(0.00039)	(0.00312)	(0.00036)	(0.00631)
Intercept		6.631 ***	7.478 ***	6.3241 *	−2.233	20.19 ***
		(1.744)	(2.339)	(3.629)	(3.080)	(7.043)
Observations		3180	1592	804	952	476
AIC		8319.6	4318.7	2110.1	3301.1	590.7
BIC		8446.9	4431.5	2203.4	3398.3	678.1

* *p* < 0.1; ** *p* < 0.05; *** *p* < 0.01. Note: Model 1 is based on the entire sample of the apartment complexes in Seoul, and subsamples for Models 2 and 3 are defined by 10 districts with apartment complexes that participate in the energy community program and the other 10 districts that do not participate. Models 4 and 5 used data from hot and cold spots, respectively. AIC is Akaike Information Criterion, BIC is Bayesian Information Criterion.

## Data Availability

The data presented in this article are available on request from the corresponding author.
